# Statistical and health economic analysis plan for a secure care hospital evaluation of manualised (interpersonal) art-psychotherapy: the SCHEMA randomized controlled trial

**DOI:** 10.1186/s13063-025-08934-3

**Published:** 2025-07-01

**Authors:** Jennifer Condie, Matthew Franklin, Katie Aafjes-van Doorn, Paula Foscarini-Craggs, Iain McKinnon, Toni Leigh Harrison, Arman Iranpour, Ania Zubala, Sophie Rose, Elizabeth Randell, Rachel McNamara, Muhammad Riaz, Simon Hackett

**Affiliations:** 1 Centre for Trials Research, Cardiff University, Neuadd Meirionnydd, Heath Park Way, Cardiff, CF14 4YS UK; 2 Sheffield Centre for Health and Related Research (SCHARR), School of Medicine and Population Health, Regent Court, The University of Sheffield, 30 Regent Street, Sheffield, S1 4DA UK; 3 New York University Shanghai, 567 West Yangsi Rd, Pu Dong Xin Qu, Shanghai, 200124 China; 4 Faculty of Medical Sciences, Population Health Sciences Institute, Newcastle University, Baddiley-Clark Building, Richardson Road, Newcastle Upon Tyne, NE2 4AX UK; 5 Cumbria, Northumberland, Tyne & Wear NHS Foundation Trust, Jubilee Rd, Newcastle upon Tyne, NE3 3XT UK; 6 Centre for Brain Sciences, Kennedy Tower. Royal Edinburgh Hospital, The University of Edinburgh, UK, Morningside Place, Edinburgh, EH10 5HF UK

**Keywords:** Secure care, Intellectual disability, MOAS, Aggression, Art-psychotherapy

## Abstract

**Background:**

The SCHEMA trial evaluates whether interpersonal art psychotherapy reduces the frequency/severity of aggressive incidents or patient distress associated with psychiatric symptoms, compared to usual care.

**Objective:**

To describe the statistical and health economic analysis plan.

**Methods:**

A multicentre, two-arm, parallel-group, single blind individually randomised controlled trial with 150 adults within NHS secure care who have borderline to mild/moderate intellectual disability. The primary outcome is the frequency/severity of aggressive behaviour, measured on the Modified Overt Aggression Scale (MOAS) 19 weeks post-randomisation, analysed using a linear mixed-effect model, adjusted for baseline MOAS and stratification by gender and psychosis diagnosis. Changes in aggressive behaviour will be evaluated using weekly MOAS scores between 19 and 38 weeks. Patient distress relating to psychiatric symptoms will be assessed using the Brief Symptom Inventory Positive Symptom Distress Index across baseline, 19, and 38 weeks. Health-related quality-of-life will be assessed using self- and proxy-reported EQ-5D three-level (EQ-5D-3L) and Recovering Quality of Life 10-item measures, the latter to estimate the ReQoL Utility Index, across baseline, 19, and 38 weeks. The self-reported EQ-5D-3L is collected using an adapted version for people with intellectual disabilities. Resource-use is collected based on secure care records, to estimate intervention and healthcare costs over 19 and 38 weeks. HRQoL and cost data will inform cost-effectiveness based on the incremental cost per quality-adjusted life year over 38 weeks.

**Discussion:**

This paper details the planned analyses and discusses recruitment challenges, sample size implications, and effect size assumptions. The plan was developed prior to database lock and unblinding to minimise analytical bias.

**Trial registration:**

ISRCTN, ISRCTN57406593. Registered on 18/01/2023.

**Supplementary Information:**

The online version contains supplementary material available at 10.1186/s13063-025-08934-3.

## Background

Aggression and violence are a cause of major problems in psychiatric and secure inpatient care [1]. An international review indicated that patients in secure care settings are likely to be more violent than those in other types of psychiatric units, with 69% of assaults against NHS staff in England occurring in mental health or intellectual disability (ID) settings [2]. It has also been suggested that patients being treated in a secure care intellectual disability ward are more likely to stay in high and/or medium secure care for longer, compared to patients on other types of secure mental health wards [3]. There is reasonable evidence of the pharmacological treatment in violence reduction where people have mental illness in forensic settings [4], and this treatment may also be likely to have a positive impact on violence in people with ID and comorbid mental illness. However, there is limited evidence for the effective use of psychotropic medication as well as non-medicinal psychotherapies for the treatment of challenging behavior in people with ID. Therefore, the development of effective interventions for people in secure care with ID and borderline intellectual functioning (BIF) is a priority.

Art psychotherapy (art therapy) is used in some NHS services to help children, young people, and adults with mental health difficulties [5, 6]. A systematic review of the clinical efficacy of art therapy among people with non-psychotic mental health disorders concluded that art therapy appeared to have statistically significant positive effects compared with usual care [7]. National practice-based guidelines have been developed for art therapy with people who have an ID [8]. Initial findings from a systematic review reported better outcomes for individual therapy compared with group-based interventions [9]. In a systematic review and meta-analysis of art therapy in forensic settings (secure care), mechanisms and positive effects of arts therapies were indicated to reduce risk and increase protective factors in individuals in forensic institutions [10].

Interpersonal Art Psychotherapy has been developed in the UK as a manualised intervention delivered by a trained art psychotherapist registered with the Health and Care Professions Council (HCPC) [11]. The SCHEMA trial aims to determine whether interpersonal art psychotherapy can reduce the frequency and severity of aggressive incidents and/or patient self-reported distress associated with psychiatric symptoms, compared to usual care. The study population includes adults within secure care who have borderline to mild/moderate ID [12].

A protocol paper describing the design of SCHEMA has previously been published [12]. This paper describes the detailed statistical and health economic analysis plan (SHEAP) for SCHEMA and specifies the statistical and health economic models for evaluation of the primary and secondary outcomes, including the covariates to be included in the adjusted analyses, and has been prepared in accordance with the published guidelines on the content of statistical analysis plans [13] (see Appendix D). In accordance with good clinical practice and to avoid outcome reporting bias [14], the SHEAP was developed before the database was locked and data analysis was initiated.

### Trial objectives

#### Primary objective

To assess the effectiveness of interpersonal art psychotherapy (compared to usual care) in reducing the frequency and severity of aggressive behaviour in adults in secure care [12].

#### Secondary objectives

1. To determine if interpersonal art psychotherapy is cost-effective compared to usual care.
2. To explore patient characteristics and psychotherapeutic processes/mechanisms within interpersonal art psychotherapy that are influential to treatment.
3. To explore the longitudinal changes in aggressive behaviour after receiving art psychotherapy.
4. To evaluate changes in patient distress related to psychiatric symptoms.

## Methods

### Trial design

Full details of the SCHEMA trial design, setting, intervention, outcomes, and ethical approvals have been published in the protocol [12]. However, we provide a summary here as follows. The trial is a multicentre, two-arm, parallel-group, single-blind, individually randomised controlled trial (RCT) of effectiveness, comparing manualised interpersonal art psychotherapy to usual care. The RCT is being conducted in 8 NHS Trusts with secure care facilities and aims to recruit 150 participants (75 per trial arm). The total trial duration is 43 months with 20 months of recruitment. The end of the trial is defined as the last participant’s last visit [12].

The intervention arm is scheduled to receive 12 (1 h each) individual interpersonal art psychotherapy sessions, delivered by a trained HCPC registered art psychotherapist, plus usual care. Participants can receive up to 3 additional sessions throughout the trial for more personalised support, adjusting for the participant’s specific communication or therapeutic needs. The sessions cover the following 7 components: (1) personal goals, (2) coping responses and self-management, (3) relationships, (4) life events, (5) interpersonal themes, (6) imagined future and (7) final review. Participants will be considered as having completed the intervention if they complete at least 5 out of the 7 components listed above.

The control arm is scheduled to receive usual care, and participants are assessed and treated by specialist professionals. The multi-disciplinary team includes psychiatrists, clinical and forensic psychologists, mental health and ID nursing staff, and Allied Health Professionals. The team uses the Care Programme Approach to coordinate and plan participant care, including risk assessments, recovery-focused care and/or positive behaviour support [15]. Participants also have access to psychotherapy/psycho-educational work, specific offence-related treatment and pharmacotherapy treatment. Participants in the control arm are offered the art psychotherapy intervention at the end of their participation in the trial.

### Ethical approval

This protocol and related trial documents were reviewed by the London-City & East Research Ethics Committee and received full approval on 13/01/2023 (REC ID: 23/LO/0026; IRAS project ID: 319,325). Informed consent was obtained from all participants prior to being entered into the trial [12].

This is a low-risk trial of non-investigational medicinal product (NIMP); therefore, a Data Safety Monitoring Board was not required. This was discussed and approved by the Trial Steering Committee (TSC). The TSC review all safety data and have met at least annually. The TSC comprises members independent of the trial, serving as chair, statistician, expert therapist, and a public member, all of whom have signed the remit and conditions outlined in the TSC Charter [12].

### Randomisation

Participants were randomly allocated to receive either art psychotherapy plus usual care (UC) as the intervention arm, or UC with delayed art psychotherapy as the control arm. Participants were allocated to the intervention and control arm in a 1:1 ratio using randomly permuted blocks of 2, 4 and 6 created in Stata (version 17). This concealed allocation and minimised the predictability of the next allocation at the end of each block. Randomisation was stratified by sex and diagnosis of psychosis to achieve balance of baseline characteristics in the two groups.

The final randomisation list was generated by the senior statistician independently, keeping the trial statistician blind to the randomisation list. Randomisation took place online through the REDCap SCHEMA database and was available 24 h a day. Treatment allocations were automatically generated once the participant’s baseline data had been entered. As this is a single blind trial, the trial statistician, health economist and research support staff completing the primary outcome assessment are kept blind to allocation. For the statistical analysis, all data cleaning, manipulation, and analysis will be carried out by the trial statistician who will be kept blind to the arms allocation.

### Sample size

The primary objective is to assess the effectiveness of art psychotherapy in reducing the frequency and severity of aggressive behavior in adult secure care as measured by the Modified Overt Aggression Scale (MOAS) [16]. Based on the results of the feasibility study [11], we assumed a minimum clinically important difference (MCID) of 5 points on the MOAS scale, a common standard deviation of 10 points in the control and intervention groups, and a correlation of 0.25 between the baseline and post-treatment MOAS scores. In addition, we may need to account for clustering by therapist, as a single therapist will apply the intervention to more than one patient. We assumed that each site would have a minimum of 2 therapists and the intraclass correlation (ICC) of the MOAS scores for the same therapist would be 1% in the intervention arm. Under these assumptions, 80% power and a type I error rate of 5%, 59 participants would be required in each arm. To account for an assumed attrition rate of 20%, this figure is inflated to a final sample size of 75 participants per arm, yielding a final sample size of 150 participants.

This would also allow the study to detect a change of 0.5 points on the Brief Symptom Inventory (BSI) Positive Symptom Distress Index (PSDI), used to evaluate changes in patient distress related to psychiatric symptoms [17]. Using an estimated SD of 0.8, the sample size calculated above would result in a power greater than 90% for the BSI comparison.

If the target recruitment (sample size) is not achieved, we will use sensitivity analysis with appropriate statistical methods as planned in the statistical analysis section. If the effect size (i.e. the mean difference of change in MOAS and its standard deviation) differs from our assumptions used in the sample size calculation, this may affect the power/sample size as shown in Table 1.

**Table 1:** Potential effects of differing effect sizes and standard deviations on the sample size

Effect size	Power	SD	Method	Correlation (baseline and follow-up)	Alpha	Sample size (per arm)	Final sample size (inc. 20% dropout)
4	0.8	10	ANCOVA	0.25	0.05	92	230
6	0.8	10	ANCOVA	0.25	0.05	41	103
5	0.8	8	ANCOVA	0.25	0.05	37	93
5	0.8	12	ANCOVA	0.25	0.05	85	213

### Framework

SCHEMA is a superiority trial as the primary objective is to assess whether interpersonal art psychotherapy is superior in terms of reducing the frequency and severity of aggressive behaviour in adults in secure care, compared to usual care.

### Timing of final analysis

No interim analyses were planned or conducted. Data collection is expected to be completed by May 2025. The data will be cleaned, and the trial database will be locked in the month following final data collection. We aim to begin the final statistical analyses in May 2025, with the goal of publishing the report in August 2025.

### Timing of outcome assessments

Outcome assessments (Table 2) are conducted at baseline (prior to randomisation), 19 and 38 weeks post-randomisation with a ± 2 weeks window.

**Table 2:** Schedule of enrolment, interventions, and assessments

Procedures	Screening	Baseline	Randomisation	Treatment phase	19 weeks	Weekly	38 weeks	Ad hoc
Informed consent	X							
Eligibility assessment	X							
LDSQ	X							
Treatment allocation			X					
Demographics		X						
Medical history		X						
Q1 HONOS (WAA or LD)	X							
Randomisation		X						
Delivery of intervention				X				
Compliance				X				
MOAS		X			X	X	X	
EQ-5D-3L self-reported LD adapted version*		X			X		X	
EQ-5D-3L Proxy Version 1		X			X		X	
Resource use					X		X	
ReQoL-10 Self Report version		X			X		X	
ReQoL-10 Proxy reported**		X			X		X	
BSI		X			X		X	
Adverse event assessments								X
Physician’s withdrawal checklist								X

*LDSQ* Learning Disability Screening Questionnaire, *Q1 HONOS (WAA or LD)* Item 1 of the Health of the Nation Outcome Scale (Working Age Adults or Learning Disabilities), *MOAS* Modified Overt Aggression Scale, *BSI* Brief Symptom Inventory

*EQ-5D-3L self-reported using LD (learning disabilities) adapted version

**ReQoL-10 Self-Report version completed by a proxy

## Statistical principles

The results will be summarised using point estimates, 2-sided 95% confidence intervals (CI) and *p*-values. A *p*-value < 0.05 will imply a test result is statistically significant. As this trial has a single primary outcome of interest, it does not require adjustment for multiplicity for the primary analysis. Adjustment for multiplicity will not be undertaken for secondary or sensitivity analyses.

### Adherence and protocol deviations

#### Adherence

Participants’ adherence to art psychotherapy is recorded as the proportion of randomised participants completing the intervention (completion of 5 out of 7 components of art psychotherapy). Intervention fidelity is recorded as the proportion of therapists adhering to the treatment manual. A therapist’s adherence to art psychotherapy is monitored during supervision sessions. Audio recorded sessions are optional and a random sample of 3 timepoints for 3 participants across 9 therapists (27% of sessions) will be blind rated using the interpersonal art psychotherapy treatment fidelity checklist, incorporating tested methods for assessing treatment fidelity (see Appendix A). Where participants do not consent to having intervention sessions audio-recorded, the number of components covered and sessions attended will be recorded as part of the week-19 follow-up. Descriptive statistics on participant adherence and intervention fidelity will be presented in a table, overall and by trial arm.

#### Deviations

A protocol deviation occurs when the participant, study coordinator or investigator fails to adhere to significant protocol requirements, including eligibility violations, deviation from intervention or non-adherence to the protocol. Protocol deviations are classified as a deviation, protocol violation or serious breach and the impact on participants’ rights, safety, wellbeing, and data integrity are classified as major, minor or no impact. We are also recording whether the deviation requires follow-up, and the PI determines if a violation results in withdrawal of a participant.

The number (and percentage) of patients with major and minor protocol deviations will be summarised by treatment group with details of the type of deviation provided. The patients that are included in the intention-to-treat (ITT) analysis dataset will be used as the denominator to calculate the percentages. Deviations that affect data integrity will be summarised in the final report.

### Analysis population

The analysis will be performed on an ITT basis; therefore, all participants who are randomised will be included in the analysis. However, if missing outcome data do not meet any appropriate assumptions such as considering the outcome was missing because the intervention was not effective, the analysis will be limited to a complete-case analysis. All protocol violations including participants who have not completed at least 5 components out of the 7 components of art psychotherapy will be considered, and we will conduct a per-protocol analysis where applicable. In addition, we will conduct a sensitivity analysis for missing outcome observations and other important variables such as psychometrics.

## Study population

### Screening data

Tables will present the following summaries (overall and by study site): the number of days recruiting, number of patients screened, number of patients recruited, number of patients recruited per day, number of screened patients not recruited, and the reason for non-recruitment.

Eligibility.

Participants are eligible for the trial if they meet the following inclusion criteria and none of the exclusion criteria apply. The number of participants falling into the exclusion criteria will be tabulated by treatment group.

#### Inclusion criteria

- An inpatient in an NHS secure hospital/unit/service with the presence of learning disability/borderline intellectual functioning indicated by either (a) meeting validated assessment criteria (recognised cognitive testing and adapting functioning assessment), or (b) a score of 57 or below on the Learning Disability Screening Questionnaire (LDSQ)
- Age 18 to 60 years
- Able to give informed consent
- A current or historic HONOS (Health of the Nation Outcome Scale) score between 1 and 4 for item 1 (overactive, aggressive, disruptive, or agitated behaviour/behavioural problems directed at others)
- The participants’ involvement in the study is supported by their responsible clinician and/or multidisciplinary team (MDT)

#### Exclusion criteria

- Unable to give informed consent
- Learning disability/borderline intellectual functioning not indicated based on a validated assessment or screening questionnaire (i.e. not meeting validated assessment criteria or a LDSQ Score > 57).
- A HONOS score of 0 for item 1
- Planned discharge within 9 months of the start of the study.
- Unstable/unmanaged psychotic symptoms requiring active assessment or treatment including medication dose titration (i.e. dose adjustment in the previous 4 weeks or with potential further dose adjustment planned for the following 4 weeks).

### Recruitment

**Fig. 1:**
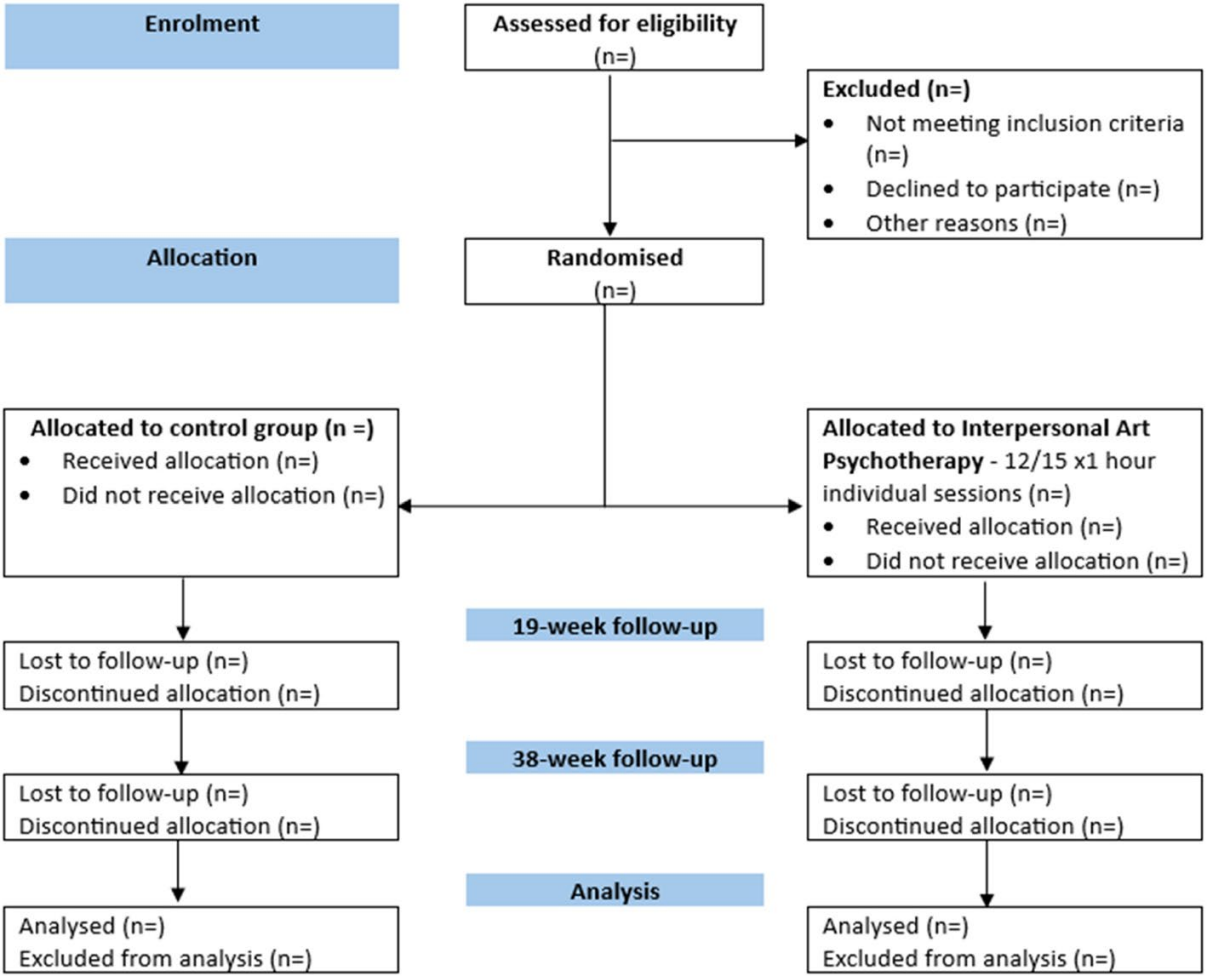
Consolidated Standards of Reporting Trials (CONSORT) diagram of the flow of participants through the trial

A CONSORT flow diagram (Fig. 1) will summarise the number of adults in secure care who were:

- Assessed for eligibility at screening
- Eligible at screening
- Ineligible at screening*
- Eligible and randomised
- Eligible but not randomised*
- Randomised to each trial arm
- Received the randomised allocation
- Did not receive the randomised allocation*
- Lost to follow-up*
- Discontinued the intervention*
- Randomised and included in the primary analysis
- Randomised and excluded from the primary analysis*
  *Reasons will be provided.

### Withdrawal/follow-up

The level of withdrawal will be tabulated and will be classified as:

- Withdrawal from trial treatment/intervention (art therapy)
- Partial withdrawal from trial-related questionnaires (will do some questionnaires)
- Full withdrawal from trial-related questionnaires (will not do any more questionnaires)
- Partial withdrawal from trial-specific follow-up visits (will attend some follow-up visits)
- Full withdrawal from trial-specific follow-up visits (will not attend any more follow-up visits)
- Withdrawal from qualitative interviews
- Withdrawal from submission of routinely collected data
- Withdrawal from audio recordings
- Withdrawal from sharing de-identified data with the research community
- Withdrawal of Consent to all the above

A participant may withdraw or be withdrawn from the trial intervention for the following reasons:

- Withdrawal of consent for treatment by the participant
- Any alteration in the participant’s condition which justifies the discontinuation of the intervention in the investigator’s opinion.
- Non-compliance (inability to follow protocol)
- Withdrawal due to a serious adverse event
- Withdrawal from control group art therapy waiting list due to mental health deterioration.
- Lost to follow-up.

A participant may be lost to follow-up for the following reasons:

- Moving outside of clinical team care
- Missing multiple visits
- Not contactable/refusing further contact.

The numbers (with reasons) of losses to follow-up (dropouts and withdrawals) over the course of the trial (baseline, randomisation, treatment phase, 19 and 38 weeks post randomisation) will be presented in the CONSORT diagram.

## Trial data

### Baseline characteristics

We are collecting demographic (age, gender, ethnic background, primary mental health diagnosis, index offence, and length of admission), medical (psychotic illness, length of diagnosis, HoNOS item 1 score, LDSQ score, full scale IQ, and other health conditions), and primary and secondary outcome (MOAS, adapted EQ-5D-3L (self-report), EQ-5D-3L Proxy Version-1 (proxy-report), ReQol-10 (self-report and proxy-report), and BSI) variables at baseline.

### Primary outcome

The primary outcome is the frequency and severity of aggressive incidents as measured by the Modified Overt Aggression Scale (MOAS) at week 19 post-randomisation.

The MOAS is completed by a research nurse or clinical support officer at baseline (prior to randomisation), and weekly from week 19 to week 38 post-randomisation, and if necessary, checking with a member of the ward team to ensure that it accurately reflects the participant’s behaviour. The MOAS is not always completed by the same research nurse/clinical support officer, so we are recording who has conducted each assessment.

The MOAS is a validated observer-rated measure that assesses the frequency and severity of aggressive incidents among people with ID over the past week (ICC = 0.93 [16]). The scale consists of four categories: verbal aggression, aggression against property, auto-aggression (against self), and physical aggression (against others). Each category will be scored using a 5-point scale (0 = no events within that category, 4 = most severe form of aggression within the category). The rater selects as many items as are appropriate. The items in each category are summed. The summed scores are multiplied by the severity weight for that category (verbal aggression × 1, aggression against property × 2, auto-aggression × 3, physical aggression × 4). The weighted scores are summed to obtain the total MOAS score, with a higher score indicating more aggressive behavior [16]. Thus, the primary outcome is a change in the MOAS score from baseline to week 19 post-randomisation.

### Secondary outcomes

1. Cost-effectiveness will be assessed based on health-related quality-of-life (HRQoL) and cost estimates. HRQoL (self-report and proxy-report) is measured using the EQ-5D-3L and ReQoL-UI, the latter derived from the ReQoL-10, recorded at baseline, 19 and 38 weeks post-randomisation. Costs are based on unit costs applied to an assessment of intervention and healthcare use recorded at 19 and 38 weeks post-randomisation.

#### EQ-5D-3L

The EQ-5D-3L has a self-report version that has been adapted to ease the burden on participants with mild to moderate intellectual/learning difficulties/disabilities (adapted EQ-5D-3L; see Appendix B). The adapted EQ-5D-3L questionnaire is completed by the participant and the EQ-5D-3L Proxy Version-1 by ward staff at baseline, 19 weeks and 38 weeks post-randomisation. The questionnaire is completed by a member of staff who is familiar with the participant’s general health; however, it may not always be completed by the same person, so we are recording who has conducted each assessment.

The questionnaire consists of two sections: the descriptive system and the visual analogue scale (VAS). The descriptive system in the adapted questionnaire comprises the following five categories aligned with the traditionally used EQ-5D-5L, each describing a different aspect of health: mobility (i.e. ‘walking about’), self-care (e.g. ‘looking after myself’), usual activities (e.g. ‘doing things I want to do’), pain/discomfort, and anxiety/depression (e.g. ‘feeling worried, sad, or unhappy’). Each category has 3 response options: no problems, some problems, extreme problems (labelled 1–3). There should be only one response for each dimension. Missing values are coded as ‘−9’. Ambiguous values (e.g. two boxes are ticked for a single dimension) are treated as missing values. This part of the questionnaire provides a descriptive profile that can be used to generate a 5-digit health state profile that represents the level of reported problems on each of the five dimensions. For example, ‘11,223’ indicates no problems with mobility and self-care, some problems with usual activities and pain/discomfort, and extreme problems with anxiety/depression. While state ‘11,111’ indicates no problems on any of the five dimensions. A utility-based (aka, preference-based) value set can be applied to the health state profile score, facilitating the calculation of quality-adjusted life years (QALYs). In this trial, we are using the EQ-5D-3L value set derived for the UK using a standardised valuation exercise, in which a representative sample of the general population is asked to place a value on EQ-5D-3L health states using the time trade-off valuation technique [18]. UK value set scores range from − 0.594 (where 0 is a health state equivalent to dead; negative values are states suggested to be worse than dead) to 1 (perfect health) [18].

The second part of the questionnaire consists of a horizontal VAS on which the nurse rates the participant’s perceived health from 0 (worst imaginable health) to 100 (best imaginable health). The participant will complete an amended horizontal VAS, rating their health from 0 to 10 [19].

#### ReQoL-10 and ReQoL-UI

The ReQoL-10 questionnaire is completed by the participant and ward staff (as a proxy) at baseline, 19 and 38 weeks post-randomisation. For the proxy-report, we have specified that at each time point both the EQ-5D-3L and ReQoL-10 should be completed by the same proxy (i.e. same member of ward staff) and where possible it should be the same proxy across time points, but we recognise that might not always be possible. We are recording who has completed each assessment.

ReQoL-10 consists of 10 mental health questions and one physical health question. Although physical health is important to the quality of life of mental health service users, it is not included in the ReQoL-10 summary score because it is distinct from mental health. Each question is scored from ‘None of the time’ to ‘Most of the time’. There are 6 positively worded questions (Q2, 4, 5, 7, 8, and 10), scored from 0 to 4 and 4 negatively worded questions (Q1, 3, 6, and 9), scored from 4 to 0. An overall summary score is calculated by summing the scores of the 10 questions. The ReQoL-10 score can range from 0 (indicating the poorest quality of life) to 40 (indicating the highest quality of life). If a single question is unanswered, the ReQoL-10 developers suggest the mean value of the other responses can be used. If more than one question is unanswered, the overall summary score cannot be calculated. If respondents give two answers to a single question, we will record the response which represents the poorer state [20].

The ReQoL-UI classification system, developed by Keetharuth et al. [21], is based on 7 ReQoL-10 items: 3 positively (ReQoL-10 items: 5, 7, 10) and 3 negatively (ReQoL-10 items: 3, 6, 9) worded mental health items, and its one physical health item. These 7 items cover 7 themes of self-reported recovery-focused quality of life: autonomy, wellbeing, hope, activity, belonging and relationships, self-perception, and physical health. The ReQoL-UI is described as having 2 overall dimensions: a mental health (6 items) and a physical health (1 item) dimension. ReQoL-UI score ranges from − 0.195 to 1, which can be used to estimate QALYs.

#### Healthcare resource use and costs

Adults within secure care, such as the SCHEMA trial participants, have limited access to external health and social care services. As such, resource use for the trial analyses is limited to those healthcare resources that are recorded on the secure care electronic care systems for the trial participants. For example, this includes attendance at a special hospital, specialist assessment, and acute psychiatric ward admission, alongside specific staff contacts and medications. This healthcare data is collected by a combination of research staff and ward staff at 19 weeks and 38 weeks post-baseline. The resource use data is recorded within a proforma resource use measure (RUM) as part of the eCRF for the previous 19-week period since baseline or last RUM data collection time-point, i.e. each RUM is focused on the previous 19 weeks since the current point of data collection. The process involves research staff checking the patient notes for what resources the patient has used based on a proforma, which they then confirm with ward staff. As such, the healthcare data represents routinely recorded data for the trial participants, rather than self-reported, which avoids certain issues with self-reported data (e.g. recall measurement error or bias) [22]. The proforma RUM is presented within Appendix C. Through discussions with the SCHEMA TMG, RUM data is not collected at baseline to limit the data collection burden at baseline. Also, through TMG discussion, baseline resource use (i.e. resource-use consumption 19 to 38 weeks pre-baseline) was perceived not to be prognostic of resource use consumption (or other trial outcomes) over the proceeding 38 weeks post-baseline, limiting the use of baseline RUM data.

Unit costs are assigned to the resource-use information collected within the RUM to calculate total costs for each type of resource use and per trial participant [23]. Unit costs are sourced from data sources relevant to the NHS within England; this includes the National Schedule of NHS costs (e.g. for secondary care costs), Personal Social Services Research Unit (PSSRU) costs of health and social care (e.g. for staff costs), and British National Formulary (BNF; e.g. for medications) [24–26].

Unit costs will be sourced for the more recent year available at the time of attaching unit costs to the resource use data (e.g. 2024/2025) with costs prior to this year inflated using an appropriate inflation index (e.g. NHS Cost Inflation Index) [25].

The costing perspective will be that of the NHS, given the limited range of external services available or required to those within secure care. A societal perspective is not considered a relevant secondary analysis for these trial participants. The frequency/severity of aggressive incidents as measured by the MOAS (as described above) at week 38 post-randomisation.

2. Longitudinal changes in aggressive behaviour are assessed weekly between 19 and 38 weeks post-randomisation using the MOAS score (as described above).
3. Patient distress attributed to psychiatric symptoms is assessed at baseline, 19 and 38 weeks post-randomisation using the Brief Symptom Inventory (BSI) Positive Symptom Distress Index (PSDI).

The Brief Symptom Inventory (BSI) is a 53-item self-report instrument designed to assess psychological symptoms over the past week. The BSI is composed of nine primary symptom dimensions (somatization, obsessive–compulsive, interpersonal sensitivity, depression, anxiety, hostility, phobic anxiety, paranoid ideation, and psychoticism). Participants rate the extent to which a specific problem has distressed them in the past 7 days, using a 5-point scale (0 = not at all to 4 = extremely). Scores are determined by summing values for each symptom dimension and then dividing by the number of items in the respective dimension. The Positive Symptom Total (PST) is the number of items with a positive (nonzero) response and the Positive Symptom Distress Index (PSDI) is the sum of the values with positive responses divided by the PST. Raw scores can be converted to standardised *T* scores, generating a profile that graphically illustrates a respondent’s current psychological symptom presentation [17]. The BSI PSDI is recorded at baseline, 19 and 38 weeks to assess changes in patient distress attributed to psychiatric symptoms.

## Statistical analyses

The summary of the analysis plan is shown in Table 3.

**Table 3:** Summary of the analysis plan

Variable	Intention-to-treat cohort
Baseline characteristics	Descriptive statistics by the trial arms
Primary outcome	• Histograms and other graphical presentation • Linear mixed-effect regression model • Homoscedastic and heteroscedastic regression models
Secondary outcomes	• Histograms and other graphical presentation • Repeated measures ANCOVA model • Linear mixed-effect models
Safety outcomes	Chi-square test
Subgroup analyses	Linear mixed-effect regression model with an interaction term
Sensitivity analyses	Multiple imputation

### Descriptive statistics: baseline and other time-points

Baseline characteristics will be summarised by trial arm using appropriate descriptive statistics.

Categorical data will be summarised by numbers and percentages. Continuous data will be summarised by means (SD) if data are normally distributed and medians (IQR) if data are skewed. Minimum and maximum values will also be presented for continuous data. Summary statistics will be visualised using appropriate graphical plots, including bar graphs, histograms, and boxplots. Baseline and outcome measures will be summarised overall and by trial arm. Tests of statistical significance will not be undertaken for baseline characteristics; rather, the clinical importance of any imbalance will be noted in the trial report. Baseline data will also be summarised for those who completed follow-up compared to those lost to follow-up (i.e. no data at weeks 19 and 38) to assess possible dropout bias.

For the economic analyses, descriptive statistics will focus on the EQ-5D-3L and ReQoL-10 item scores and utility scores, QALYs, resource-use and costs across all resource-use items. Intervention resource-use and costs will also be presented separately to the downstream resource-use and cost descriptive statistics, i.e. the resource-use and cost associated with interpersonal art psychotherapy. All descriptive statistics will be provided as stratified by trial-arm, at/up to each data collection time-point (i.e. at baseline, at/up to 19 weeks and 38 weeks), and across all time-points (i.e. over 38 weeks) as appropriate.

### Analysis of primary outcome

The aim of the primary analysis is to examine the effectiveness of art psychotherapy in reducing the frequency and severity of aggressive incidences compared to usual care, as measured by the change in MOAS score from baseline to week 19 post-randomisation. The distribution of the MOAS scores will be assessed graphically using histograms and the mean (SD) or median [IQR] of the change in score from baseline to week 19 will be tabulated overall and by trial arm (art psychotherapy versus usual care). The change of primary outcome from baseline to week 19 will be assessed graphically using a scatter plot.

The primary outcome analysis will be performed on an ITT basis. Initially, we will use a linear mixed-effect regression model [27, 28], with the week-19 MOAS score as the dependent variable and the randomisation group as the independent variable, adjusted for the baseline MOAS score, random effect of the variable indexing strata based on gender and diagnosis of psychosis. In addition, we anticipate potential clustering in the intervention arm due to therapists. We will examine this by the ICC from the linear mixed-effect model and if there is a considerable amount of heterogeneity in the primary outcome associated with therapists, we will adjust the effect estimate for the partial clustering due to therapists. For this, both partially homoscedastic and heteroscedastic regression models will be explored, and we will report the results as appropriate. The homoscedastic model assumes between-cluster variability is only present in the intervention arm and the individual level variation is the same in both arms. The heteroscedastic model assumes between-cluster variability is only present in the intervention arm but allows different individual level variation in intervention and control arms [29, 30]. From the initial model(s), we will present the estimated difference in MOAS score at week-19 for the intervention group compared to the control group with 95% CI.

### Analysis of secondary outcomes

Secondary outcomes include the cost-effectiveness of art psychotherapy (EQ-5D-3L and ReQoL-UI), longitudinal changes in aggressive behavior (weekly MOAS score) and changes in patient distress relating to psychiatric symptoms (BSI PSDI). The distribution of the secondary outcomes will be assessed graphically using histograms and boxplots. Depending on the score distributions, the mean (SD) or median [IQR] will be tabulated overall and by trial arm (art psychotherapy versus usual care).

For the longitudinal changes in aggressive behavior using the weekly MOAS score from week 19 to week 38, the changes will be graphically explored by plotting the mean weekly MOAS scores. As an exploratory analysis, a repeated measures ANCOVA model will be fitted which will include treatment group and the baseline value as the independent variables, and with the weekly MOAS score as the dependent variable. Analyses will be adjusted for stratification based on sex and diagnosis of psychosis.

Further secondary outcomes analyses will be performed as ITT analyses. The secondary outcomes of EQ-5D-3L, ReQoL-10 and BSI PDSI will be recorded at three time points (baseline, 19 and 38 weeks post randomisation). We will use separate linear mixed-effect models with week 19 and 38 scores (EQ-5D-3L, ReQoL-10 and BSI PDSI) as the dependent variables and the interaction of randomisation group with time (week 19, week 38) as the independent variable, adjusted for relevant baseline scores, random effect of the variable indexing strata based on gender and diagnosis of psychosis, and a random effect of clustering (by therapist). We will present the estimated difference in EQ-5D-3L/ReQoL-10/BSI PDSI scores at week 19 and 38 for the intervention group compared to the control group with 95% CI.

### Covariate adjustment

If we have enough statistical power, analyses to compare interpersonal art psychotherapy and UC usual care will include the relevant baseline outcome measures.

### Assumption checking

Before the analyses, the distributions of the primary outcome (MOAS score) and secondary outcomes (EQ-5D-3L, ReQoL-10, weekly MOAS score and BSI PDSI) will be examined using histograms, QQ plots, boxplots and the Shapiro–Wilk test, to ensure they meet the distributional assumptions of mixed-effect linear regression models/ANCOVAs.

### Alternative methods if distributional assumptions not met

If the data does not meet the distributional assumptions of linear mixed-effects regression, the data will be transformed if possible (e.g. log with base 10). If transformations do not improve the distributions of scores, non-parametric/semi-parametric statistical methods or dichotomising the outcome scores will be considered. Non-parametric methods include generalised estimating equations (GEE) [31–33], quantile regression [34], and bootstrapping [35]. If a dichotomous/categorical version of an outcome is used, mixed-effect logistic [36] or multinomial logistic regression [37] will be considered.

### Subgroup analyses

The following subgroup analyses will be exploratory only. After the main analysis, we will report our key findings by age groups, gender, ethnicity and primary mental health diagnosis. We will investigate whether the treatment effect varies by primary mental health diagnosis or level of learning disability using the LDSQ score. We will repeat the primary analysis and additionally include an interaction term between allocation and primary mental health diagnosis and (in a separate analysis) between allocation and LDSQ score.

We will also conduct an analysis of reliable change for therapy completers (5 out of 7 components of art psychotherapy) using Brief Symptom Inventory Positive Symptom Distress Index (BSI PSDI) scores, allowing for comparison against clinical and non-clinical norms. The Reliable Change Index (RCI) is a psychometric criterion used to evaluate whether a change over time of an individual score (i.e. the difference in BSI PSDI score between baseline and 38 weeks) is considered statistically significant [38]. Firstly, we will identify the RCI on the BSI from baseline (B) to 38 weeks follow-up (F). Participants will be categorised as responders if they see improvement from B to F, neutral if their scores do not change, and deteriorators if their scores worsen. We will repeat the process for MOAS score. To explore the differences between therapy responders and non-responders, we will conduct the primary analysis and additionally include an interaction term between allocation and therapy respondence (responder/neutral/deteriorators). We will explore the demographic/clinical data for the responders and non-responders to compare the profiles.

### Sensitivity analyses

Due to the slow rate of accrual, it may not be possible to achieve the recruitment target of 75 participants per arm in the recruitment timeframe. In addition, we may not be able to achieve the projected effect size. If we observe any lack of statistical power, we will use bootstrap techniques to compute the bootstrap confidence intervals for the effect estimates and report them.

The analyses will be performed on an ITT basis, meaning all participants randomised to art psychotherapy or usual care will be included in the analysis. During data entry, validations have been written into the system to minimise the amount of missing data, however, missing data may still occur. The frequency and percentage of missing values will be reported overall and by arm.

Missing data will be investigated for cause and extent, and multiple imputations with the assumption of missingness at random (MAR) will be considered. Analyses will be conducted to assess the assumption of MAR and identify baseline variables to be used in the imputation models. Missing observations in the primary and secondary outcomes will be replaced by imputed values using the predictive mean matching method in chained equations [39, 40] of linear regression. At least 20 datasets will be created for the imputation of each outcome, and the imputation-specific estimates for the effect of intervention on the primary and secondary outcomes will be combined using Rubin’s rules [41].

These analyses will be considered as sensitivity analyses, as the analyses will be conducted with missing observations replaced by the imputed values and the results will be compared with the complete case and ITT analyses.

### Safety

The following table provides the definitions used in safety reporting (Table 4):

**Table 4:** Definitions used in safety reporting

Term	Definition
Adverse event (AE)	Any untoward medical occurrence that impacts the intervention, day to day activities, or requires medical intervention in a participant or clinical trial participant administered an intervention which is not necessarily caused by or related to that product
Serious adverse event (SAE)	Any adverse event that - · Results in death · Is life-threatening* · Required hospitalisation or prolongation of existing hospitalisation** · Results in persistent or significant disability or incapacity · Consists of a congenital anomaly or birth defect · Other medically important condition***
Serious adverse reactions (SARs)	Any SAE occurring in a clinical trial participant for which there is a reasonable possibility that it is related to the intervention
Suspected unexpected serious adverse reactions (SUSARs)	A SAR, the nature and severity of which is not consistent with the Reference Safety Information (RSI) for the intervention

*The term ‘life-threatening’ in the definition of serious refers to an event in which the trial participant was at risk of death at the time of the event or it is suspected that used or continued use of the product would result in the subjects death; it does not refer to an event which hypothetically might have caused death if it were more severe

**Hospitalisation is defined as an inpatient admission, regardless of the length of stay, even if the hospitalisation is a precautionary measure for continued observation. Pre-planned hospitalisation, e.g. for pre-existing conditions which have not worsened, or elective procedures, does not constitute an SAE

***other events that may not result in death, are not life-threatening, or do not require hospitalisation, may be considered as an SAE when, based upon appropriate medical judgement, the event may jeopardise the participant and may require medical or surgical intervention to prevent one of the outcomes listed above

For the purposes of this trial, incidences of self-harm are also considered SAEs. The frequency (percentage) of adverse and serious adverse events will be tabulated overall and by trial arm and compared using a chi-square test.

## Psychometric analyses

Psychometric assessment for this study focuses on post-hoc quantitative analyses of the SCHEMA trial’s primary and secondary outcome measures, particularly to inform the choice of preference-based measure which may be the most ‘appropriate’ for the economic evaluation (see the ‘Economic evaluation’ and ‘Discussion’ sections) and therefore conducted before starting the economic evaluation. In this case, we are specifically interested in the primary outcome measure for the SCHEMA trial (i.e. MOAS score) and those measures which can be used to produce utility scores used to estimate QALYs (i.e. EQ-5D-3L and ReQoL-UI, self-reported and proxy-reported); the BSI PSDI will also be included, but the primary comparison is with the MOAS.

In terms of psychometric properties, the focus will be on the construct validity and responsiveness of the utility scores, as the psychometric properties specified of interest to NICE within their technology appraisal methods guide [42]. Such psychometric analyses have been used to support economic evaluations previously [43, 44].

### Construct validity focussed on convergent and known-group validity

Construct validity assesses the extent to which a measure reflects HRQoL differences hypothesised to exist. We assess construct validity in relation to convergent and known-group validity.

Convergent validity assesses the relationship between measures, proposed here to be based on correlation analyses and locally weighted scatterplot smoothing (LOWESS) techniques. For example, Spearman’s rank absolute correlation strength (ACS) coefficient and associated p-value are proposed as a non-parametric test (with the actual method chosen post hoc based on the measures’ score distributions) which indicates the degree to which instruments are measuring related factors [45]. LOWESS can complement the correlation analyses as a form of non-parametric regression which plots a line of central tendency between two variables on a scatterplot, thereby visualising their general relationship across the possible score ranges without making assumptions about the actual relationship [46].

Known-group validity assesses the extent to which instrument scores differ between groups that are expected to differ, measured using Cohen’s d standardised absolute effect sizes (AES i.e. the difference in mean scores between two adjacent severity subgroups divided by the standard deviation of scores for the milder of the two subgroups) [45, 47]. For example, this will include stratification of the trial participants at each time point based on the MOAS MCID threshold of 5 points, regardless of trial arm. The non-parametric Kruskal Wallis test complements assessing AES to suggest if there is a statistically significant difference between the two (or more) known groups.

### Responsiveness

Responsiveness is important in economic evaluation as any change in health must be reflected by a change in utility/preferences and a subsequent change in QALYs. To measure responsiveness, we will examine floor (worst possible score) and ceiling (best possible score) effects, which affect the ability of the measure to detect deterioration or improvements in health, respectively. We will also examine the magnitude of change in scores over time as a crude indicator of responsiveness; however, we will cross-reference change in measure scores against changes on the MOAS (i.e. those achieving an MCID of 5 points or not).

### Additional considerations: responder perspective and face validity

There are additional considerations which can’t be assessed quantitatively but are stated here as they will be considered narratively. As the MOAS is completed by site research-staff, whereas the EQ-5D-3L and ReQoL-10 are completed by the trial-participant and ward staff (proxy), these different reporter perspectives will be narratively considered within the psychometric analyses [48, 49]. Additionally, from a self-reported perspective by the trial-participant, it is perceived that the adapted EQ-5D-3L will have face validity whereas the ReQoL-10 may not and this impacts on the trustworthiness of the self-reported scores. These considerations will inform the interpretation and description of the psychometric results.

## Economic evaluation

Cost-effectiveness analysis via cost per quality-adjusted life year (QALY) is recommended internationally, including by the National Institute for Health and Care Excellence (NICE) for England and Wales [42]. Therefore, in adults within secure care who have borderline to mild/moderate ID, we will calculate the mean incremental cost per QALY of interpersonal art psychotherapy compared to usual care over 38 weeks, from a healthcare perspective.

Cost-effectiveness will be inferred when comparing our incremental cost-effectiveness results to a relevant cost-effectiveness threshold, such as thresholds from £0 (whereby cost savings are preferred to QALY gains) up to £50,000 per QALY, with specific attention to £20,000 to £30,000 per QALY thresholds as suggested by NICE [42].

The primary QALY-based analyses will use the self-reported, adapted EQ-5D-3L, albeit its use has implications/complications (see the ‘Discussion’ section). To calculate QALYs, the area-under-the-curve (AUC) will be calculated using the data collected for the EQ-5D-5L and ReQoL-UI at baseline, 19 weeks, and 38 weeks [50].

The economic evaluation will be conducted on an ITT basis, based on guidance by Hunter et al. [50] and Franklin et al. [23]. To calculate mean incremental costs and QALYs, both observed and (regression) adjusted mean values will be estimated and compared [44]. Choice of regression model for the adjusted analyses will follow the choices also made for the statistical analyses of the primary outcome (e.g. generalised linear-mixed models) as will the choice of other covariates, while also considering the relevant prognostic nature of the covariate to the economic evaluation outcomes of interest (e.g. baseline utility for QALYs) [50].

Incremental cost-effectiveness ratios (ICERs) will be calculated based on incremental costs over incremental QALYs as the estimated mean difference between the two trial arms. The ICER represents the cost per QALY of the intervention-arm compared to control-arm which will be compared to a relevant cost-effectiveness threshold to infer ‘cost-effectiveness’ or not.

Bootstrapping will be used to estimate standard errors and confidence intervals and generate cost-effectiveness planes and cost-effectiveness acceptability curves (CEACs). Cost-effectiveness planes will be used to present the dispersion of the estimated ICERs following resampling with replacements as part of the bootstrapping process, over 5000 resampled iterations. The CEACs will be used to visualise and report on the probability of cost-effectiveness across a range of cost-effectiveness threshold values (e.g. up to £50,000 per QALY).

Missing data, sensitivity, and sub-group analyses will align with that proposed for the primary statistical analyses for which there is also an interest related to the economic evaluation.

## Statistical software

Statistical analysis will be carried out using Stata (StataCorp LLC, TX, USA) version 17 or higher.

## Discussion

This paper presents the detailed SHEAP for SCHEMA trial, which is a two-arm, parallel-group, single-blind, individually randomised controlled trial of effectiveness, comparing manualised interpersonal art psychotherapy to usual care. The main objective is to assess the effectiveness and cost-effectiveness of interpersonal art psychotherapy (compared to usual care) in reducing the frequency and severity of aggressive behaviour in adults in secure care. This SHEAP is aimed to evaluate the effect of intervention on primary and secondary outcomes with an adjustment for the pre-planned covariates in mixed effect linear regression models. We will analyse data on an intention-to-treat basis and use datasets generated by multiple imputations using chained equations to assess the effect of missing observations on the analyses if required. In addition, we will conduct a per-protocol analysis which will include only those participants who adhere to the intervention.

The SHEAP will minimise the risk of outcome reporting bias as having a predefined plan will minimise the risk of data-driven analyses. We strengthen our results by replacing missing observations with imputed values from multiple imputations by chained equations, facilitating calculation of unbiased estimates, standard errors and confidence intervals.

It is worth mentioning here that the proxy EQ-5D-3L and ReQoL-10 questionnaires completed by the ward staff at baseline, 19 and 38 weeks post-randomisation will be completed by a member of staff who is familiar with the participant’s general health; however, it may not always be completed by the same person, so we will record who has conducted each assessment. The variability in who completes the questionnaire could introduce measurement bias or inter-rater variability, which might affect the reliability and validity of the proxy EQ-5D-3L outcome. The ICC could be calculated to quantify the degree of agreement between nurses, and an exploratory sensitivity analysis could include the research nurse as a random effect in the primary analysis.

The study has found it harder to recruit participants than anticipated and has led to a slower recruitment rate. An 80% power was approved by the NIHR and resulted in a reduction in the required sample size. However, it is anticipated that the study may not achieve the current target sample size of 75 participants per arm. Due to the smaller sample size, we could have wider confidence intervals, reflecting greater uncertainty about the true effect size; as such, results may need to be interpreted with this in mind. However, we have planned to use bootstrap techniques to compute the bootstrap confidence intervals for the effect estimates and report them.

For the economic analysis, the primary HRQoL measure for estimating QALYs is the self-reported, adapted EQ-5D-3L. The rationale is that the self-reported, adapted EQ-5D-3L best aligns with the current NICE methods for technology appraisal guidelines and has been developed specifically for people with learning disabilities. However, there are complications within this patient population that might mean that although NICE prefers the EQ-5D-3L and the adapted version has face validity in the patient population (i.e. those with learning disabilities), it might not be the most appropriate measure to use for the economic analysis; therefore, psychometric analyses are proposed to inform the preferred outcome for the economic analyses. The use of psychometric analyses means the choice of outcome measure for the economic analysis will be chosen post-hoc (albeit, the psychometric analyses will be completed before conducting the economic evaluation); however, as post-hoc choices can allude to data/outcome mining (e.g. making the results fit the preferred outcome), this post-hoc choice does not change the primary outcome for the economic analyses which will remain the adapted, self-reported EQ-5D-3L. Rather, the psychometric analyses results will be published alongside the economic analyses, with the economic analyses results, discussion and conclusion informed by the psychometric analyses to suggest which outcome measure has the best psychometric performance and therefore may be the more ‘appropriate’ outcome for the economic analysis, despite the primary outcome remaining the adapted, self-reported EQ-5D-3L.

Finally, this analysis plan is comprehensive, which includes a detailed statistical and health economic analysis plan coupled with an appropriate psychometric evaluation of the measures. We believe that application of this SHEAP will reduce the risks of outcome reporting bias and data-driven results, facilitating an unbiased evaluation of the trial data and supporting confidence in our findings.

## Trial status

The final version of this analysis plan was approved by the trial steering committee on the 04/02/25. The first patient was enrolled on the 05/05/2023, with the last participant expected to complete follow-up on the 12/04/2025 (± 2 weeks).

## Conclusion

The SCHEMA trial aims to assess the effectiveness and cost-effectiveness of interpersonal art psychotherapy in reducing the frequency and severity aggressive behaviour in adult secure care compared to usual care. This paper describes the planned statistical and health economic analyses used in the SCHEMA trial in order to minimise the risk of data-driven results and outcome reporting bias. Any deviations from the analysis plan will be reported and justified in the final report.

## Supplementary Information

Supplementary Material 1. Appendix A – Interpersonal Art Psychotherapy Treatment Fidelity Checklist. Appendix B – Adapted EQ-5D-3L Questionnaire. Appendix C – Week 19 and 38 resource use questionnaires. Appendix D: Statistical Analysis Plan (SAP) Guidance [13].

## Data Availability

Not applicable.

## Abbreviations

SCHEMA: Secure Care Hospital Evaluation of Manualised (interpersonal) Art-psychotherapy
NHS: National Health Service
MOAS: Modified Overt Aggression Scale
BSI PSDI: Brief Symptom Inventory Positive Symptom Distress Index
HRQoL: Health-related quality-of-life
EQ-5D-3L: EQ-5D three-level
ReQoL-10: Recovering Quality of Life 10-item
ReQoL-UI: Recovering Quality of Life Utility Index
QALY: Quality-adjusted life year
ID: Intellectual disability
BIF: Borderline intellectual functioning
HCPC: Health and Care Professions Council
SHEAP: Statistical and Health Economic Analysis Plan
RCT: Randomised controlled trial
UC: Usual care
MCID: Minimum clinically important difference
ICC: Intraclass correlation
SD: Standard deviation
LDSQ: Learning Disability Screening Questionnaire
HONOS: Health of the Nation Outcome Scale
WAA: Working Age Adults
LD: Learning Disability
CI: Confidence interval
ITT: Intention-to-treat
MDT: Multidisciplinary team
CONSORT: Consolidated Standards of Reporting Trials
IQ: Intelligence quotient
VAS: Visual analogue scale
RUM: Resource use measure
eCRF: Electronic case report form
TMG: Trial management group
PSSRU: Personal Social Services Research Unit
BNF: British National Formulary
PST: Positive Symptom Total
ANCOVA: Analysis of covariance
IQR: Interquartile range
QQ: Quantile-quantile
GEE: Generalised estimating equations
RCI: Reliable Change Index
MAR: Missing at random
AE: Adverse event
SAE: Serious adverse event
SAR: Serious adverse reaction
SUSAR: Suspected unexpected serious adverse reaction
NICE: National Institute for Health and Care Excellence
LOWESS: Locally weighted scatterplot smoothing
ACS: Absolute correlation strength
AES: Absolute effect size
AUC: Area under the curve
ICER: Incremental cost-effectiveness ratio
CEAC: Cost-effectiveness acceptability curve
NIHR: National Institute for Health Research
